# Accelerating Pythonic Coupled-Cluster Implementations: A Comparison Between CPUs and GPUs

**DOI:** 10.1021/acs.jctc.3c01110

**Published:** 2024-02-02

**Authors:** Maximilian H. Kriebel, Paweł Tecmer, Marta Gałyńska, Aleksandra Leszczyk, Katharina Boguslawski

**Affiliations:** Institute of Physics, Faculty of Physics, Astronomy, and Informatics, Nicolaus Copernicus University in Toruń, Grudziadzka 5, 87-100 Toruń, Poland

## Abstract

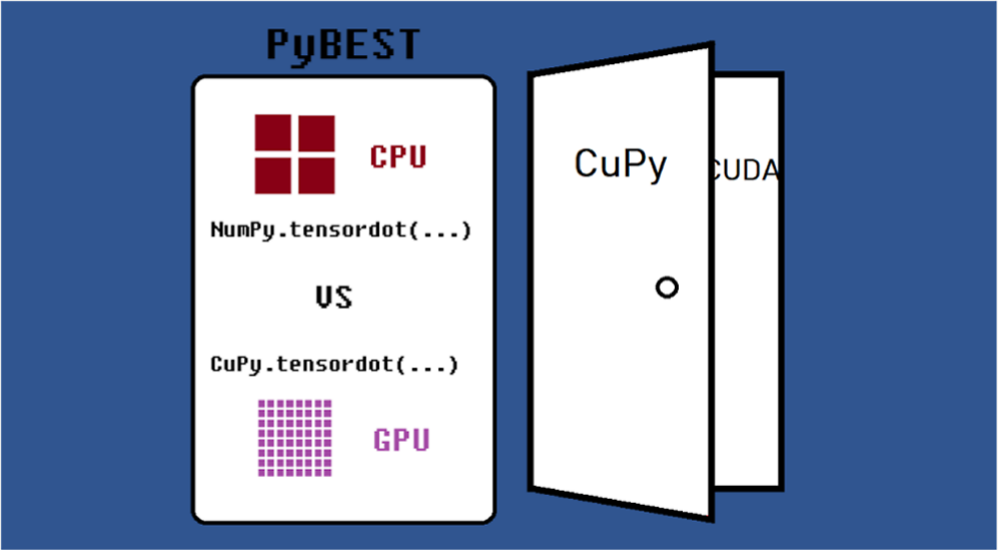

In this work, we benchmark several Python routines for time and memory requirements to identify the optimal choice of the tensor contraction operations available. We scrutinize how to accelerate the bottleneck tensor operations of Pythonic coupled-cluster implementations in the Cholesky linear algebra domain, utilizing a NVIDIA Tesla V100S PCIe 32GB (rev 1a) graphics processing unit (GPU). The NVIDIA compute unified device architecture API interacts with CuPy, an open-source library for Python, designed as a NumPy drop-in replacement for GPUs. Due to the limitations of video memory, the GPU calculations must be performed batch-wise. Timing results of some contractions containing large tensors are presented. The CuPy implementation leads to a factor of 10–16 speed-up of the bottleneck tensor contractions compared to computations on 36 central processing unit (CPU) cores. Finally, we compare example CCSD and pCCD-LCCSD calculations performed solely on CPUs to their CPU–GPU hybrid implementation, which leads to a speed-up of a factor of 3–4 compared to the CPU-only variant.

## 1 Introduction

A critical job of a graphics card is to compute projections of three-dimensional objects to a 2D surface using linear algebra. These calculations can be performed in parallel effectively, meaning that multiple small mathematical operations such as multiplication and addition can be performed simultaneously. For this reason, graphics processing unit (GPU) development mainly focuses on massively increasing the number of computing cores. While a central processing unit (CPU) may have up to 128 cores on the high end, GPUs already have up to 16 000 cores (like NVIDIA RTX 4090).

In scientific computing, the distribution of calculations among multiple CPU cores and multiple nodes is a standard practice.^1−5^ Due to its inherently parallel structure, linear algebra operations can be calculated in parallel and, therefore, efficiently offloaded to the GPU.^6−10^ The first to report independently that the internal hugely parallel structure of GPUs can be misused, not to compute graphics, but to be utilized in quantum chemistry were Yasuda^11^ and Ufimtsev and Martinez,^12^ respectively. Today, the potential of GPUs for nongraphics-related computations is widely understood and often used to accelerate quantum chemistry methods like density functional approximations,^13^ Hartree–Fock theory,^14,15^ Møller–Plesset perturbation theory,^16−18^ coupled cluster (CC) theory,^3,8,19−21^ and the evaluation of effective core potentials^22^ to name a few. The NVIDIA compute unified device architecture (CUDA) API offers a C++ interface to utilize GPU computing power.^23^ A relatively quick way to interact with this API is, for example, CuPy,^24^ a Python library which internally uses CUDA routines for the utilization of NVIDIA GPUs and since release 9.0 also ROCm for the utilization of AMD GPUs. CuPy brings a lot of attention from Python-based software developers as its interface is highly compatible with NumPy^25^ and allows even for a drop-in replacement in some particular cases. Although some fine control is sacrificed when exploiting libraries like CuPy, third-party libraries are quickly accessible without the necessity of in-detail backend control and, therefore, a very convenient and efficient way to probe graphics processor utilization in the first place.

In this work, we present benchmark results comparing the timings of the bottleneck tensor contractions present in CC calculations restricted to at most double excitations, where Cholesky vectors approximate the electron repulsion integrals.^26^ Specifically, we perform several flavors of these bottleneck contractions on a CPU by using Python libraries and benchmark their resource requirements. These contractions are calculated with, for instance, NumPy’s^25^ tensordot and einsum routines or opt_einsum^27^ on CPUs. Moreover, we elaborate on exporting these operations on a GPU exploiting CuPy’s tensordot routine.^24^ All the calculations are performed in the open-source PyBEST software platform.^28−32^ The unique features of PyBEST include pCCD-based methods for ground- and excite-state calculations,^33^ a quantum entanglement and correlation analysis,^34−38^ and the design of a modular tensor contraction engine (TCE).^28−32^ In the following, we focus on the TCE and its interface to the CuPy library. These characteristics make PyBEST different from other Python-based quantum chemistry software packages.^39−45^

This work is organized as follows. In Section 2, we briefly discuss the main bottleneck operations in CC calculations. Section 3 scrutinizes several Python-based strategies to compute the CC vector function. In Section 4, we examine the PyBEST tensor contraction engine. A GPU implementation exploiting the CuPy library is summarized in Section 5. Numerical results and the assessment of the GPU to CPU performance are presented in Section 6. Finally, we conclude in Section 7.

## 2 The CC Ansatz

Our starting point is the CC ansatz^46−51^
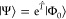
1where  is the cluster operator and |Φ_0_⟩ some reference wave function like the Hartree–Fock determinant. In this work, we will consider, at most, double excitations in the cluster operator, that is, . We do not consider single excitations explicitly as the bottleneck operations are due to the  excitation operator. Furthermore, we will work in the spin-free representation, with spin-free amplitudes, and the CC equations are spin summed. In this picture, the double excitation operator takes on the form
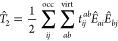
2with the CCD amplitude *t*_*ij*_^*ab*^ and  being the singlet excitation operator
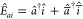
3where  indicate electron creation operators for α (β) electrons, while  are the corresponding annihilation operators. The above sum runs over all occupied (occ) and virtual (virt) orbitals for the chosen reference determinant |Φ_0_⟩.

The scaling-determining step in the CCD amplitude equations is associated with the following term
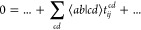
4

Summation over the indices *i*, *j*, *a*, *b* is implied, which results in the formal scaling of the CCD equations of . In the above equation, ⟨*ab*|*cd*⟩ are the electron-repulsion integrals (ERI) in Physicist’s notation. To reduce the storage of the full ERI, they can be approximated by Cholesky decomposition^26,52^
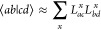
5where *x* indicates the summation over the elements of the Cholesky vectors. Since we work with real orbitals with eightfold permutational symmetry of the ERI, both Cholesky vectors *L*_*ac*_^*x*^ and *L*_*bd*_^*x*^ are identical. Then, the CC excitation amplitudes are optimized iteratively by rewriting the rate-determining step of the vector function  of iteration *n* of the optimization procedure
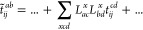
6including a third summation index running over the Cholesky vectors (ignoring the summation over the fixed indices *i*, *j*, *a*, *b*). Thus, formally, the complexity increases to . However, the formal scaling can be reduced to  (if *x* > *o*^2^) or  (if *v* > *o*^2^) by defining suitable intermediates. While the former scheme creates an intermediate of size *xo*^2^*v*^2^, the latter one features an intermediate array of size *v*^4^. Note that the dimension of *x* depends on the chosen threshold of Cholesky decomposition. For decent to tight thresholds (around 10^–6^), we have *x* ≈ 5(*o* + *v*).

## 3 Pythonic Implementations of the CC Vector Function

To solve the CC amplitudes iteratively, we must evaluate the vector function of eq 6 in each iteration including all of its terms. Thus, we will focus on the bottleneck operations of the corresponding CCD vector function. Within a Python-based implementation, we can utilize various Python libraries to perform summations efficiently through an implementation which minimizes function calls.^28,29,39,40,44,45,53^ To that end, Python-based implementations are becoming more popular among scientists. We should stress, however, on the fact that implementations based on compiled languages (such as C++ or FORTRAN) have more potential in delivering efficient computer codes. However, it comes at the cost of more laborious implementations. Based on the chosen routines, these summations (in the following called contractions) can be performed in one shot or sequentially, creating several intermediates to boost efficiency and reduce resource requirements or even enabling out-of-the-box parallelization. In the following, we will scrutinize different variants to evaluate eq 6 as Pythonically as possible, focusing solely on Python features and libraries. Specifically, we focus on the NumPy routines einsum and tensordot, the opt_einsum package, and a GPU implementation exploiting CuPy’s tensordot feature.

### 3.1 einsum and opt_einsum

The possibly easiest and most straightforward way of avoiding nested for-loop implementations when dealing with tensor contractions is to refer to NumPy’s einsum routine, which evaluates the Einstein summation convention on a sequence of operands. Using numpy.einsum, many—albeit not all—linear algebraic operations on multidimensional arrays can be represented in a simple and intuitive language. With an increasing version number, additional features and improvements have been incorporated into the numpy.einsum function. One crucial parameter is the optimize argument, which allows control over intermediate optimization. If set to “optimal”, an optimal path of the contraction in question will be performed. Another possibility is to exploit the numpy.einsum_path function to steer the order of the individual contractions in the most optimal way.

An effort to improve the performance of the original numpy.einsum routine led to the development of the opt_einsum package.^27^ It offers several features to optimize numpy.einsum significantly, reducing the overall execution time of einsum-like expressions. For instance, it automatically optimizes the order of the underlying contraction and exploits specialized routines or BLAS.^54^ opt_einsum can also handle various arrays from different frameworks such as NumPy, Dask, PyTorch, Tensorflow, or CuPy, to name a few. Furthermore, the optimization of numpy.einsum has been passed upstream of the original numpy.einsum project. Some of opt_einsum’s features can hence be utilized by numpy.einsum modifying the optimization option. Since opt_einsum features more up-to-date algorithms for complex contractions, we will focus on the opt_einsum.contract function to evaluate the Einstein summation convention on a sequence of operands, typically containing three multidimensional input arrays. opt_einsum.contract represents a replacement for numpy.einsum, where the order of the contraction is optimized to reduce the overall scaling (and hence increase the computational speed-up) at the cost of several intermediate arrays. To steer the memory limit and prevent the generation of intermediates that are too large, opt_einsum.contract offers the memory_limit parameter to provide an upper bound of the largest intermediate array built during the tensor contraction.

Thus, the bottleneck contraction in eq 6 can be straightforwardly evaluated using, for instance, opt_einsum.contract as follows
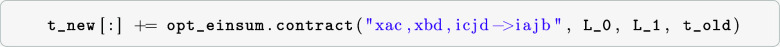


In the above code snippet, t_new indicates the vector function of iteration *n* + 1, t_old the current approximate solution (iteration *n*) of the CCD amplitudes, and L_0 (L_1) is the Cholesky vector of eq 5. Note that  are stored as a four-dimensional NumPy array t_new[i,a,j,b].

### 3.2 tensordot

An alternative routine to perform a tensor contraction is the tensordot function offered in NumPy^55^ and CuPy.^24^ It efficiently computes the summation of one (or more) given index (indices). It allows for saving memory by de-allocating intermediate arrays and explicitly defining the path of the complete tensor contraction. Furthermore, tensordot makes use of the BLAS^54^ API and features a multithreaded implementation when linked against the proper libraries like OpenBLAS,^56^ MKL,^57^ or ATLAS.^58^ A contraction along one axis of two arrays A and B
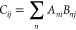
translates into
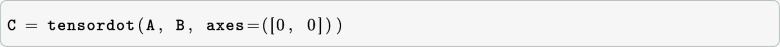


Similarly, tensordot can contract (that is, sum over) two or more axes in one function call, where
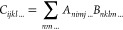
translates into
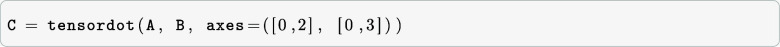


Note, however, that tensordot allows for contracting only two operands at a time. Thus, to evaluate the term in eq 6, a sequence of tensordot calls must be performed where suitable intermediates are created. One possibility is to contract the Cholesky vectors to create an intermediate of dimension *v*^4^, which is then passed to a second tensordot call generating the desired output.
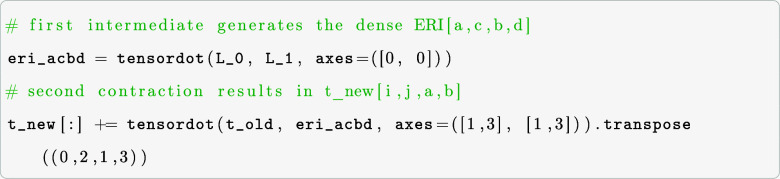


Note that tensordot does not reorder the axis. In that case, we need to transpose the intermediate result to match the shape of the output array (the vector function). However, generating a *v*^4^ intermediate of the ERI might be prohibitive regarding memory requirements for larger systems. Other possibilities lead to even larger intermediates. For instance, contracting L_0 with t_old yields a multidimensional array of size *xo*^2^*v*^3^, which is smaller than the eri_acbd intermediate if *xo*^2^ ≪ *v*, where *x* is a prefactor depending on the threshold of the Cholesky-decomposed ERI. This prefactor is typically challenging to determine a priori. Nonetheless, for computationally feasible problems, the condition *xo*^2^ ≪ *v* is rarely satisfied, making the first contraction path computationally more efficient, in terms of memory.

A less elegant, albeit computationally cheaper, way to use all of the benefits of the tensordot function is to introduce one for-loop to iterate over one axis. If we choose to loop over the second axis of L_0, we generate intermediates of at most *v*^3^
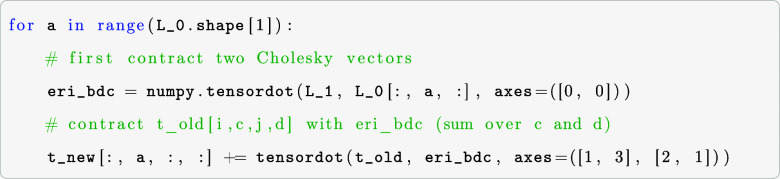


The following will refer to the contraction path above as our numpy.tensordot routine. Note, however, that this is not purely a numpy.tensordot computation, but an iterative call of the numpy.tensordot method to calculate eq 6 to prevent the creation of *v*^4^ intermediate tensors.

## 4 A Modular Implementation of Tensor Contractions

We have implemented and benchmarked the performance of the above-mentioned tensor contraction routines in PyBEST.^28^ Specifically, PyBEST is designed as a modular toolbox where the wave function-specific implementations are decoupled from the linear algebra operations.^28,29^ In an actual calculation, the logic in choosing the optimal tensor-contraction scheme is as follows
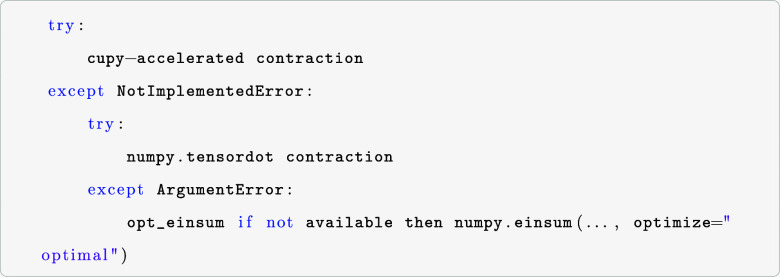


The first try statement enforces that the selected tensor contractions are performed on the GPU, if available. If bottleneck-specific contractions are not supported or a CUDA-ready GPU is unavailable, a numpy.tensordot call is performed. Since numpy.tensordot supports only specific contractions featuring nonrepeating indices (that is, repeated indices have to be summed over in numpy.tensordot), an opt_einsum call is performed or, if opt_einsum is not available, an optimized numpy.einsum function call is made instead. Another faster possibility is to default to opt_einsum calls instead of (batched) numpy.tensordot routines based on the expected memory requirements and available resources. The corresponding tensor contraction operation is written using the Einstein-summation convention of the numpy.einsum module; that is, all mathematical operations use an input and output string, where repeated indices are summed over. That allows for one unique notation of mathematical operations independent of the underlying representation of the tensors, especially the ERI. As an example, the bottleneck contraction in eq 6 translates to the string “abcd,ecfd–>eafb”, where the first part (abcd) corresponds to the ERI (in their dense representation, which PyBEST internally translates to xac,xbd if Cholesky-decomposed ERI are used; vide infra), the second part (ecfd) to the doubles amplitudes, while the output string (eafb) indicates the order of the output indices of the vector function. Internally, this string is further decoded according to the notation used in the employed LinalgFactory instance, a dense or Cholesky-decomposed representation. If a dense representation of tensors is chosen, the string remains as is. For Cholesky-decomposed ERI, the input argument associated with the Cholesky instance (here “abcd”) is translated to the internal Cholesky representation, that is, “xac,xbd.”

Since numpy.tensordot only supports a summation over two multidimensional arrays at a time, we need to divide a tensor contraction containing more than two operands into appropriate intermediates. Such partitioning can be fully automated, exploiting the numpy.einsum_path function. It proposes a contraction order of the lowest possible cost for an einsum expression, taking into account the creation of intermediate arrays. The resulting tensordot_helper function has the following logic
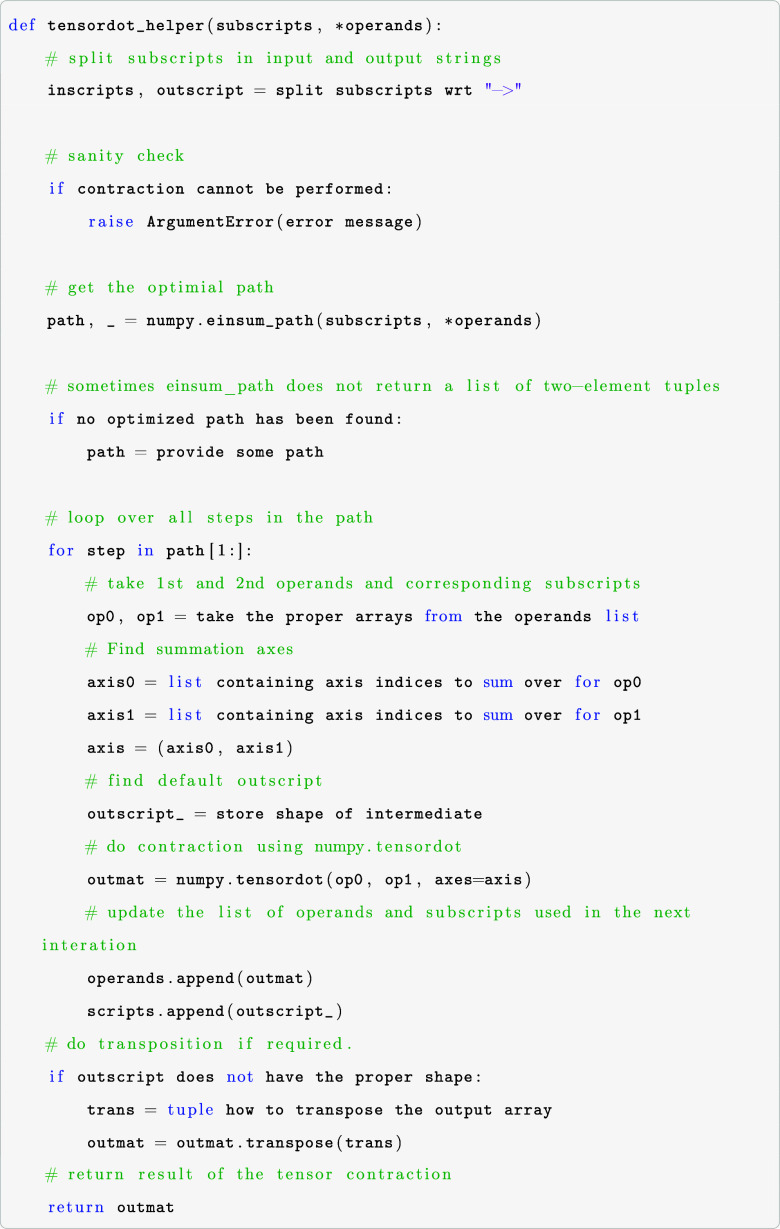


The subscripts argument is a string specifying the contraction using the numpy.einsum notation, while all multidimensional input arrays are stored in the operands argument. We assume that the ERI corresponds to the leading subscripts. If a tensor contraction cannot be performed, we transition to opt_einsum or numpy.einsum(..., optimize = “optimal”) and an ArgumentError is raised. In the case of intermediates that are too large, the tensordot_helper function is replaced by a function call containing selected hand-optimized tensor contraction operations. The implemented flow of contraction operations (CuPy–numpy.tensordot–opt_einsum/numpy.einsum) allows for optimal usage of computational resources, hardware, and multithreaded implementations. The reasons for the proposed operational flow are scrutinized in Section 6. When the performance of the libraries used is improved in future releases, the order of the contraction flavors can be adjusted to maximize efficiency without significantly interfering with the underlying source code in each wave function module.

In the following, all benchmark calculations adhere to the contraction flow mentioned above, if not stated otherwise. That is, most tensor contractions are performed using the numpy.tensordot routine, while the bottleneck contractions are outsourced to the GPU.

## 5 CuPy and Batch-Wise Computations

**Figure 1:**
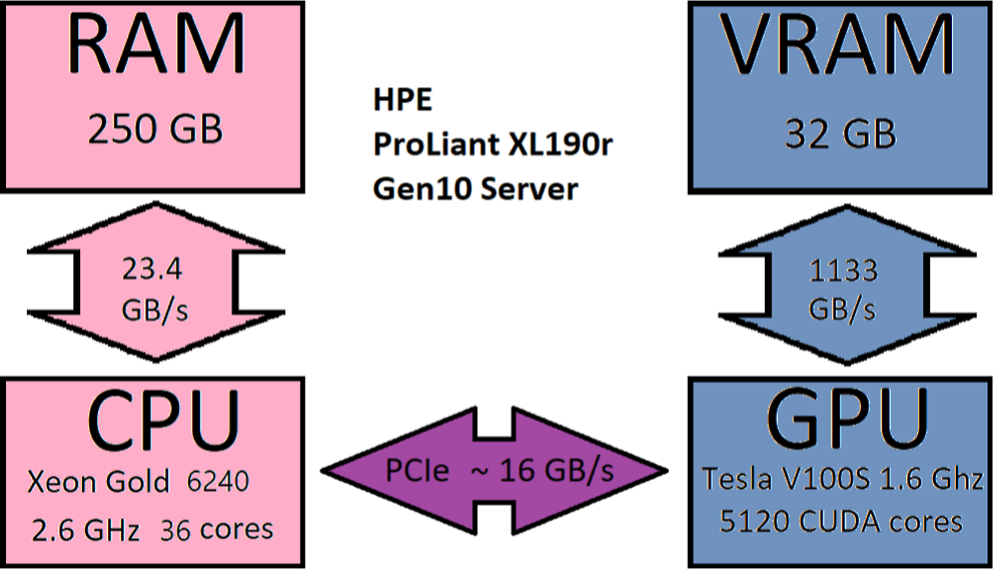
Schematic picture of RAM, VRAM, GPU, and CPU and the data transfer rate between the components.

While, of course, the most performance one could achieve by designing a method specifically for the hardware to be calculated on, we focus on accelerating the mathematical bottleneck operations by exporting the contractions in question to the GPU. As proof of the concept, we exploit CuPy^24^ for GPU-accelerated computing. Specifically, it is written as a drop-in replacement for NumPy.^55^ For medium- or large-sized molecules, we have to consider the size of the multidimensional arrays present in eq 6 and, therefore, the amount of data that needs to be processed and transferred to the video RAM (VRAM). The principle of memory and processor communication is shown in Figure 1. Due to their size, the underlying multidimensional arrays might be too big to be transferred, processed on the GPU, and transferred back. Thus, a generic implementation, where a NumPy implementation is recycled as a CuPy implementation by replacing, for instance, numpy.einsum with cupy.einsum and taking into account host to device and device to host operations, is impracticable or even impossible.

To calculate contractions on the GPU for realistic molecules and large basis sets, it is necessary to do the computations in batches. In our batch-wise computing approach, the arrays on which the operations will be performed must be copied to the VRAM in chunks. To maximize performance and utilize the massive number of CUDA cores, the size of the chunks has to be chosen as big as possible so that as few as possible cycles are needed. To achieve reasonably sized chunks of data to be processed in batches, we multiply the number of elements with their corresponding element size (in bytes) and sum up the required storage space for each multidimensional array involved. If too large, the arrays are split, and the necessary memory is checked again. This process is repeated until the split arrays fit into the VRAM. An example that shows the splitting, allocations, and de-allocations is shown in the following code example for the contraction xac,xbd,ecfd–>eafb
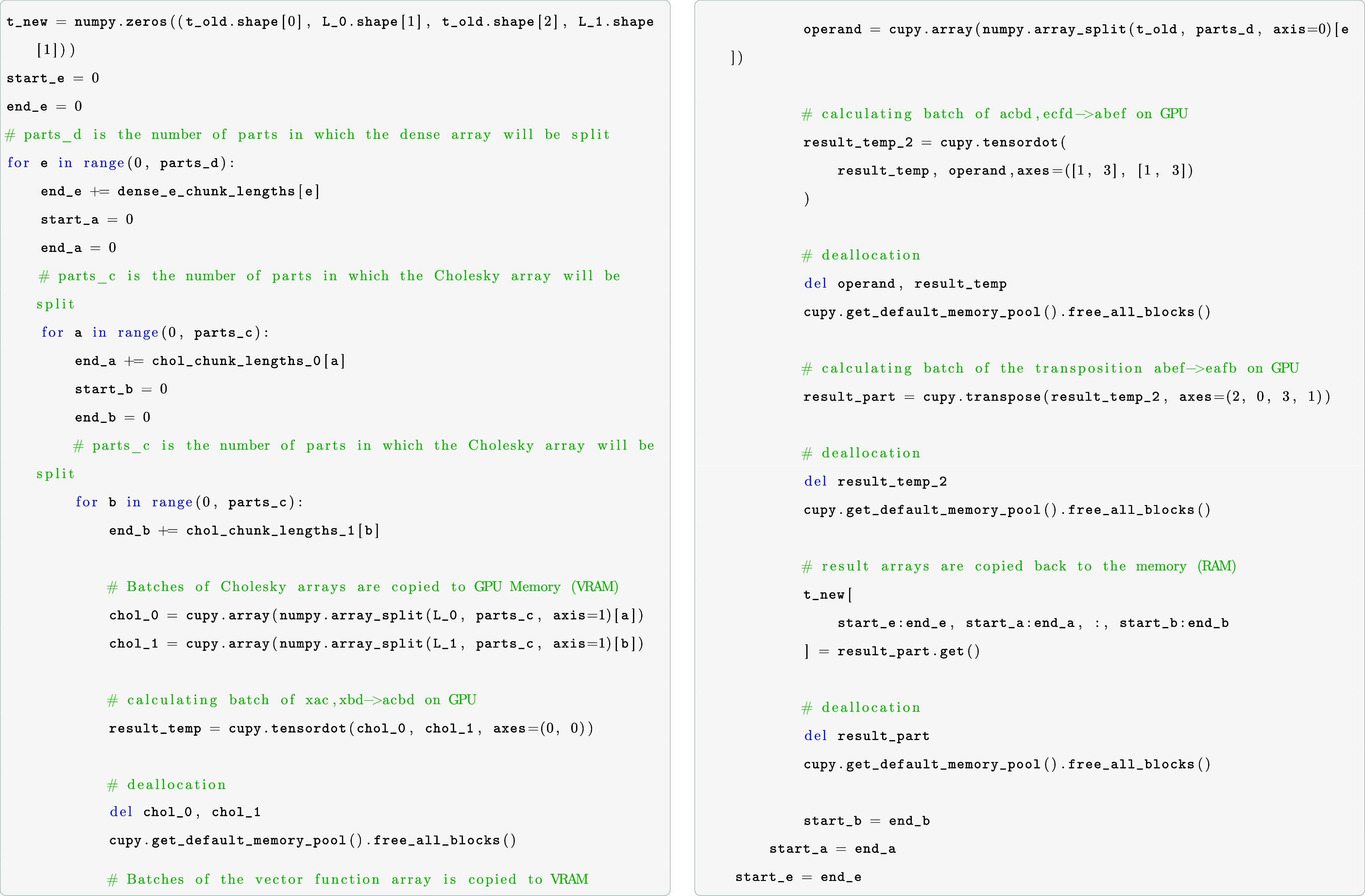


## 6 Numerical Results

### 6.1 Software Specifications and Computational Details

**Figure 2:**
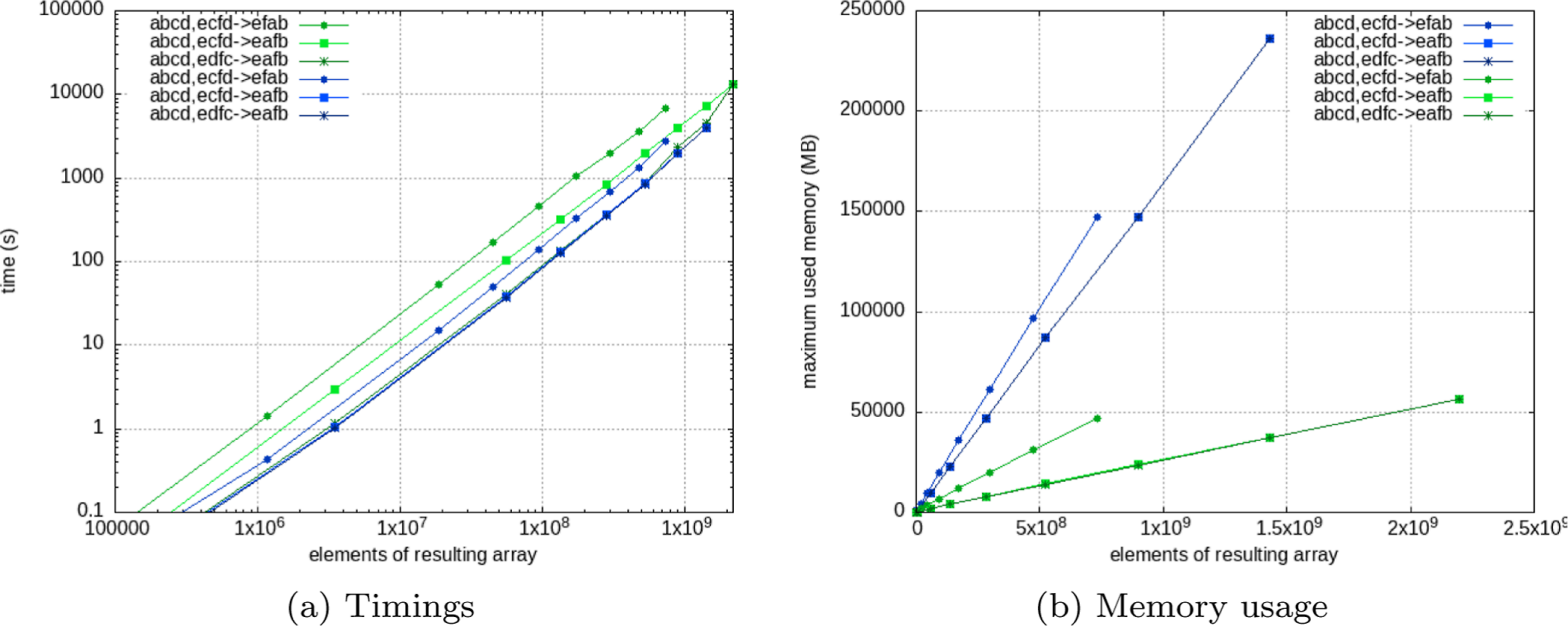
Timings (a) and memory usage (b) of numpy.tensordot and opt_einsum with respect to the size (number of elements) of the largest input array. In both plots, the greenish colors show tensordot and bluish colors show opt_einsum computations using 1 CPU core.

All calculations were performed on the CentOS 7 operating system using, if not mentioned otherwise, CUDA compilation tools v12.1.105, intelpython v3.9 (referred to as Python in the following), Intel OneAPI v2023.1, and CuPy v12.0.0. Furthermore, we employed NumPy v1.23.5, opt_einsum v3.3.0, and PyBEST v1.4.0dev, which is available on Zenodo^30^ or PyPi^32^ (the released v1.3.1 includes the CuPy features presented in this work). The hardware on which the computations were performed is gathered in Section S1 of the Supporting Information. Note that for the benchmark data shown in Figure 2, we used Intel Python 3.7, Intel OneAPI v2021.3, PyBEST v1.3.0, and NumPy v1.21.2.

The molecular structure of the L0 dye was optimized in the ORCA 5.0.3 software package^59−62^ using the B3LYP^63,64^ exchange–correlation functional and the cc-pVTZ basis set.^65^ The resulting structural parameters are provided in Section S2 of the Supporting Information. That molecular structure is subsequently used in orbital-optimized pair CC doubles (pCCD^33,66−70^) calculations, pCCD augmented with the linearized CC singles and doubles (pCCD-LCCSD) correction,^71^ and in the conventional CC singles and doubles (CCSD) approach, as implemented in the PyBEST software package.^28,29^ In all PyBEST calculations for the L0 dye, we utilized the Cholesky linear algebra factory with a threshold of 10^–5^ for the ERI. In all benchmark calculations concerning timings and memory requirements, the Cholesky vectors are random arrays, where we assume a size of 5*K*^3^ with *K* corresponding to the number of basis functions. This roughly corresponds to a Cholesky cutoff threshold of 10^–5^ in the actual molecular calculations.

### 6.2 Comparison Between CPU- and GPU-Accelerated Implementations

To be able to make assumptions about the benefit of offloading computations to the GPU, it is reasonable to study how effectively different functions described in Section 3 perform compared to each other. In the following, the computation times of numpy.tensordot, opt_einsum, and their CPU multicore processing behavior are investigated and compared with CuPy’s tensordot. As mentioned above, a very handy way for implementing tensor contractions is numpy.einsum or opt_einsum, which features a similar syntax. We should note that although numpy.einsum and opt_einsum have similar performance if two operands are contracted with each other, this is not the case anymore if the list of operands contains several multidimensional arrays. In the latter case, opt_einsum may be superior to numpy.einsum in terms of computing time by several orders of magnitude, primarily due to dynamical optimization of contraction paths, the parallelization of the underlying lower-level opt_einsum routines, and efficient batch operations.^72^

Thus, we only show benchmark results for opt_einsum in this work. Furthermore, we investigate three tensor contractions, namely “abcd,ecfd–>eafb”, “abcd,edfc–>eafb”, and “abcd,ecfd–>efab”. The first contraction (“abcd,ecfd–>eafb”) corresponds to the bottleneck term of eq 6, while the second one is the associated exchange term, which reads
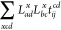
7

Although the exchange part of the formal bottleneck contraction is not present when working in a spin-free representation, we benchmark this contraction for reasons of completeness, in case a spin-dependent implementation is sought. The third contraction “abcd,ecfd–>efab” recipe is used in two additional terms of the CCSD vector function. These are one term involving an intermediate 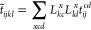
8which is an operation of  complexity, and another one comprising the ERI of 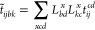
9with computational complexity of . Note that exporting eq 8 to the GPU is merely a byproduct of eq 9 as both contractions can be written using the same subscript.

Compared to the for-loop version of numpy.tensordot, the internal optimization leads to a speed-up of a factor of 2 as indicated by Figure 2a. The reason we chose numpy.tensordot as the workhorse of all tensor contractions instead of opt_einsum (or numpy.einsum), is the severe disadvantage of opt_einsum regarding memory efficiency. This drawback becomes evident in the peak memory usage displayed in Figure 2b. The internal optimization and the creation of intermediate arrays (for speed-up) lead to *a* factor of 3 faster-growing memory consumption. That disqualifies opt_einsum as a generic option primarily because we do not want to and often cannot constrain the code to smaller problem sizes. Therefore, we employ numpy.tensordot as the default contraction flavor if Nvidia CUDA is not available. We should note that opt_einsum features other arguments that limit the memory peak to a specific size. However, this feature comes at the cost of computing time. Specifically, a user-defined limit of the memory peak significantly deteriorates the speed of the numerical operations, which renders opt_einsum impractical for large-scale tensor contractions.

**Figure 3:**
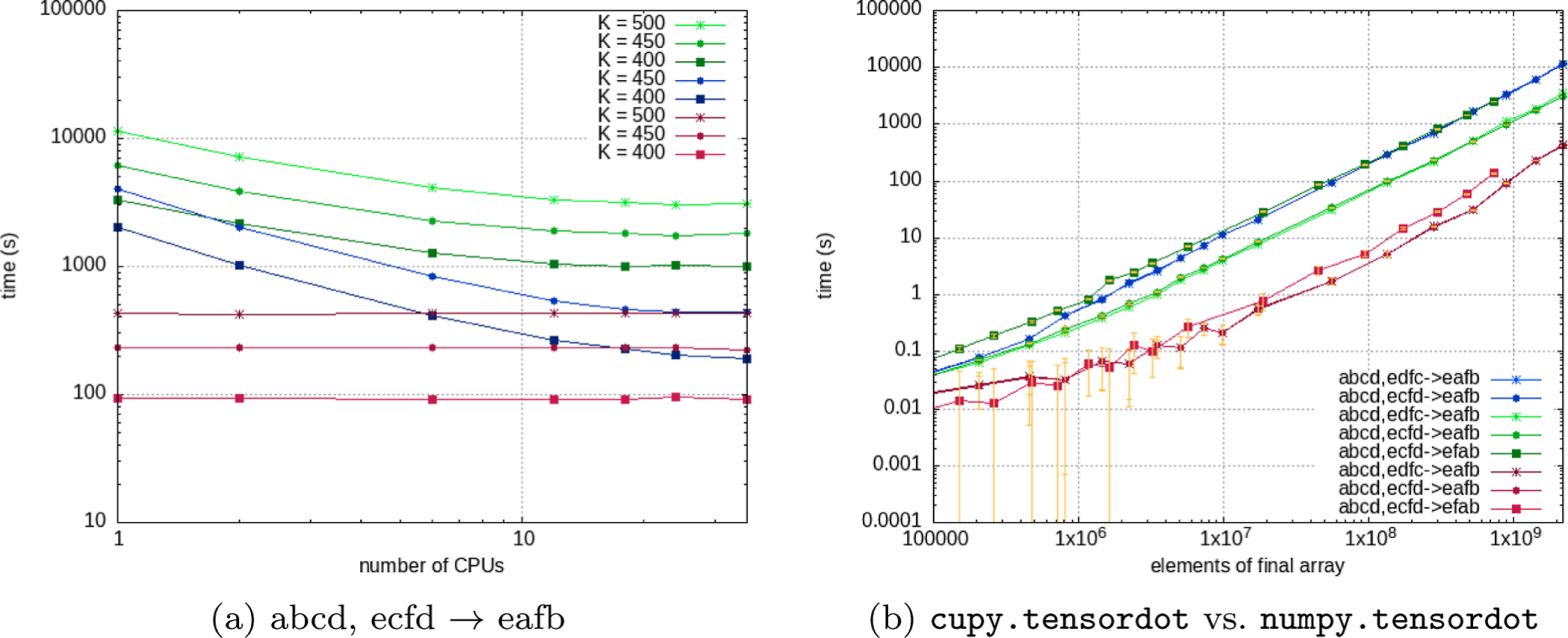
Computing times for CPU and GPU-accelerated implementations. (a) Timings for the bottleneck contraction abcd,ecfd–>eafb. The greenish colors show numpy.tensordot, the bluish colors correspond to opt_einsum, while reddish colors indicate cupy.tensordot results. *K* is the total number of basis functions. The CuPy results are shown as a guide for the eye and use all available GPU cores. (b) Comparison of GPU and CPU computing times for different basis set sizes *K*. The numpy.tensordot results for calculations with 1 CPU core are shown in bluish colors and for 36 CPU cores in greenish colors. The reddish colors show the cupy.tensordot results. The error bars in orange are determined for an average of 10 runs and show the standard deviation. For the number of elements of the final array, see the description in the main text and Table 1.

Figure 3a summarizes timings for computations of the contraction “abcd,ecfd–>eafb” (or “xac,xbd,ecfd–>eafb” if the Cholesky vectors are explicitly mentioned) plotted over the number of CPU cores for various tensor contraction flavors and problem sizes *N*. The greenish colors show numpy.tensordot, the bluish colors opt_einsum, and reddish colors indicate cupy.tensordot timing results for different numbers of CPU cores. Specifically for Figure 3a, the problem size is given by *N* = *o*^2^*v*^2^ because we investigate the following problem setup with dimensions (*xv*^2^, *xv*^2^, *o*^2^*v*^2^ → *o*^2^*v*^2^), where *v* and *o* are the number of virtual and occupied orbitals, respectively. Their sizes have been set according to the relation  and *o* = *K* – *v*, where *K* is the total number of basis functions. We should mention that the data for *K* = 500 using opt_einsum could not be obtained due to technical problems, as the memory consumption/memory peak exceeds the available physical memory of the computing node. Overall, the opt_einsum calculations are a factor 3 faster than the corresponding numpy.tensordot variants but limited by their memory peak. However, the for-loop-based numpy.tensordot variant is roughly a factor of 2 slower compared to the contraction scheme where the *v*^4^ intermediate is formed. Thus, opt_einsum and numpy.tensordot are comparable in performance, with the former being modestly faster. Note that the PyBEST project is constantly improved and tested, which means that we are also looking for a more efficient numpy.tensordot CPU implementation, utilizing what is learned from the cupy.tensordot implementation for the GPU, and vice versa. Efficient batching for better memory usage and more effective parallelization are two examples.

Figure 3a further highlights that the benefit of performing a computation on a larger number of cores shrinks very rapidly. The timings reach a plateau at about 8 to 10 cores. This data points to a decreased parallel efficiency of the CPU-based numpy.tensordot and opt_einsum routines. Note, however, that the investigated numpy tensordot scheme comprises one outer for loop, which additionally reduces parallel efficiency. Judging by the numbers, the contraction functions do not really benefit from the usage of more than 10 cores, with 4 cores being the most reasonable amount, assuming the computing time is limited. The bigger the basis set or problem size, the more benefit one gets from utilizing more cores. The cupy.tensordot timings are also shown in Figure 3a for a direct comparison (using all GPU cores). We should note that they are independent of the number of CPU cores (as the mathematical operations are performed on the GPU) and faster than both the NumPy and opt_einsum alternatives. Figure 3b compares the required computing times of CPU and GPU-accelerated contractions. The results corresponding to GPU and CPU timings are taken as an average of over 10 runs. Furthermore, the CPU data was obtained from computations exploiting 1 and 36 CPU cores, respectively. Figure 3b contains three different data sets, namely, one for each investigated contraction “abcd,ecfd–>eafb”, “abcd,edfc–>eafb”, and “abcd,ecfd–>efab”. Note that the notation is internally translated to the Cholesky vectors by replacing the “abcd” part of the string with “xac,xbd”. Thus, we always have three input operands in each tensor contraction operation. We should stress that the timings corresponding to all three contraction subscripts are very close to each other, making them almost indistinguishable from each other on the plot. For reasons of comparability, the *x*-axis shows the problem size, which is given by the size of the resulting (output) tensor. Table 1 summarizes the different problem sizes with respect to the contraction subscript, namely, *N* = *o*^2^*v*^2^ for “abcd,ecfd–>eafb” and “abcd,edfc–>eafb” and *N* = *o*^3^*v* for the contraction “abcd,ecfd–>efab”, respectively. The standard deviation for the timings of larger problem sizes is about 1% of the average value, while it increases to approximately 10% for smaller problem sizes. The elements of the arrays used to perform the benchmark calculations were randomly generated. The larger standard deviation for smaller problem sizes and very short time measurements in the order of 10^–2^ are often attributed to small changes in the workload of the operating system.^73^ Calculation time of 0.1% is used for host-to-device transfer; therefore, we do not consider PCIe a bottleneck. All in all, we observe an order of magnitude reduction of computing time for the 3 different bottleneck tensor contractions encountered in the CCSD working equation when utilizing the CuPy implementation computed on the NVIDIA Tesla V100S PCIe 32GB (rev 1a) compared to our NumPy implementation computed on 36 CPU cores.

**Table 1:** Problem Sizes *N* Determined for Different Basis Set Sizes *K* for the Selected Contractions Benchmarked in This Work^a^

	*N*			
*K*	300	400	500	
abcd,ecfd–>eafb	284 765 625	900 000 000	2 197 265 625	*o*^2^*v*^2^
abcd,edfc–>eafb	284 765 625	900 000 000	2 197 265 625	*o*^2^*v*^2^
abcd,ecfd–>efab	94 921 875	300 000 000	732 421 875	*o*^3^*v*

a The last column indicates the size of the output array, while  and .

### 6.3 A Case Study—A Sensitizer Molecule

**Figure 4:**
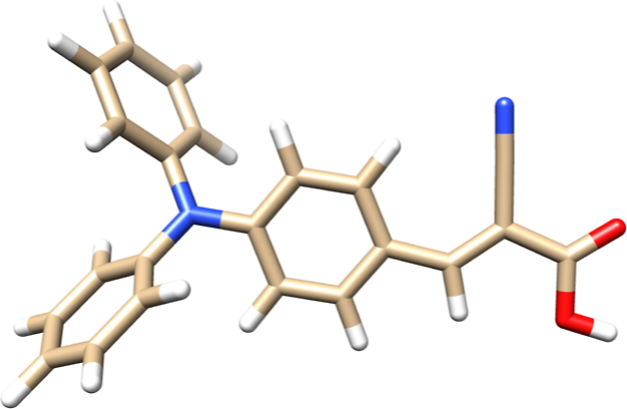
Visualization of molecular structure of the L0 dye relaxed at the B3LYP/cc-pVTZ level of theory.^75^

To check the overall performance in actual chemical problems, we perform example calculations with and without GPU acceleration. Still, the focus is on bottleneck operations. Specifically, we will scrutinize the impact on the overall computing time when just a given set of contractions are offloaded to the GPU. 2-Cyano-3-(4-*N*,*N*-diphenylaminophenyl)-*trans*-acrylic acid, commonly referred to as L0 dye (see Figure 4),^74^ has been chosen as the candidate for this performance test. This organic dye was designed to be a potential sensitizer in dye-sensitized solar cells, making it a viable alternative to ruthenium-type dyes. The size of the L0 dye allows us to benchmark the Python-based hybrid CPU–GPU implementation for a chosen parameter set.

We tested our CCSD and pCCD-LCCSD implementations by exploiting a cc-pVDZ basis set in our benchmark calculations. All calculations were performed with 36 CPU cores. We should stress that only the 3 bottleneck operations mentioned above are offloaded to the GPU, while the remaining terms in the CC vector function (or the CC iterations step) are performed on the CPU. Table 2 compares different CPU timings in seconds for cupy.tensordot to the timings of our numpy.tensordot variant. As highlighted in Table 2, one iteration step of the vector function takes around 2600 s on the CPU, while the corresponding function requires only 770 s to be evaluated in the case of the hybrid CPU–GPU variant. Note that the average iteration step time shown in Table 2 includes the evaluation of the vector function, the update of the CC amplitudes (using a quasiperturbation-based Newton step exploiting the DIIS algorithm), and the evaluation of the CC energy expression, which is similar for the CPU and hybrid CPU–GPU implementation as those operations are not offloaded to the GPU. The difference in the computing times between the CPU and CPU–GPU implementation of the vector function is the time used by the contractions that were offloaded to the GPU, namely, around 1950 s for the bottleneck contractions per CC iteration step compared to the pure CPU variant. In contrast, the bottleneck contractions offloaded to the GPU require only about 123 s per CC (vector function) iteration step, which is a speed-up of approximately a factor of 16. Comparing the resulting iteration times for the vector function evaluation of the CPU-based numpy.tensordot implementation to the CPU–GPU hybrid variant, 2600.68/766.98 ≈ 3.4, we obtain a total speed-up of approximately a factor of 3. Furthermore, while the bottleneck contractions require about 74% of the computing time per iteration step on the CPU, it drops to approximately 16% of the computing time per iteration using a CPU–GPU hybrid approach.

**Table 2:** Timings [s] and Speed-Up Factors for the CPU and Hybrid CPU–GPU Implementation for Selected CC Calculations of 2-cyano-3-(4-*N*,*N*-diphenylaminophenyl)-*trans*-acrylic acid (Referred to as “L0”) Using a cc-pVDZ Basis Set (444 Basis Functions) with 89 Occupied Orbitals^a^

		CCSD		pCCD-LCCSD	
		NumPy	CuPy	NumPy	CuPy
timings	average iteration step (CPU + GPU)	2754.21	918.25	2692.91	813.80
	average iteration step (CPU)	2754.21	795.28	2692.91	680.00
	average iteration step (GPU)		122.98		133.80
	vector function step (CPU + GPU)	2600.68	766.98	2541.80	662.38
	vector function step (CPU)	2600.68	644.00	2541.80	528.58
	vector function step (GPU)		122.98		133.80
	bottleneck contractions	1956.68	122.98	2013.23	133.80
speed-up	average iteration step	3.0	3.3		
	bottleneck contractions	16.0	15.0		
	vector function step	3.4	3.8		

a Iteration step time indicates the time [s] required for one CC iteration step. It contains the evaluation of the vector function, the update of the CC amplitudes, and the evaluation of the CC energy expression. All timings correspond to differences in epoch times. Average iteration step: mean value for the time of one CC iteration averaged over four steps. Vector function step: time of the CPU/GPU part of the vector function averaged over four steps. Bottleneck contractions: time for all bottleneck contractions investigated in this work, that is, those exported to the GPU, averaged over 4 steps.

We observe similar speed-ups for the pCCD-LCCSD method. We should note that our generic GPU implementation also offloads the contraction “abcd,cedf–>aebf” to the GPU in addition to the bottleneck operation “abcd,ecfd–>eafb”. The third bottleneck operation “abcd,ecfd–>efab” corresponds to disconnected  terms and does not show up in the pCCD-LCCSD vector function. This additional tensor contraction corresponds to a term of . As expected, the evaluation time of the pCCD-LCCSD vector function takes less time compared to the CCSD implementation, as we exclude the majority of the disconnected terms. Offloading to the GPU reduces the computing time for the selected bottleneck operations from about 2000 to 133 s, which corresponds to a speed-up factor of approximately 15. The overall speed-up for one evaluation of the pCCD-LCCSD vector function drops to a factor of 2541.80/662.38 ≈ 3.8. On average, the bottleneck contractions take about 20% of the computing time of the vector function per iteration step for the CPU–GPU hybrid implementation, while the corresponding time increases to 80% for the CPU variant, which is similar to our CCSD example.

We conclude that offloading the slowest contraction of the form of eq 6 to the GPU already leads to a significant acceleration, shifting the bottleneck to another set of terms in the CC working equations. Additional speed-up can be obtained by offloading other speed-determining tensor contractions to the GPU.

### 6.4 Comparison to the Bare CUDA Implementation

**Figure 5:**
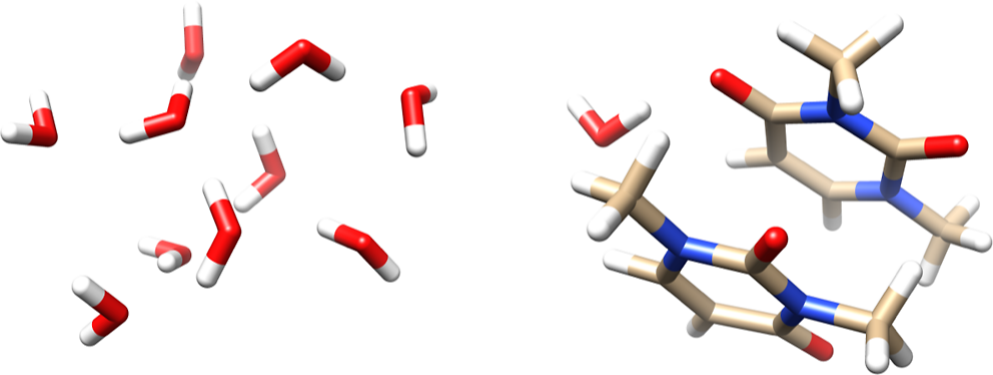
Molecular structures of (H_2_O)_10_ (left) and (mU)_2_H_2_O (right) visualized with UCSF Chimera.^75^ XYZ coordinates taken from ref (77).

In this subsection, we assess the performance of our Python-based GPU acceleration exploiting the CuPy library for a direct CUDA implementation. Specifically, we compare our PyBEST’s GPU implementation with the TeraChem^76^ benchmark data for the (H_2_O)_10_ and (mU)_2_H_2_O molecules.^77^ The molecular structures of the investigated systems are listed in Figure 5. Table 3 collects the numerical data from PyBEST and TeraChem. We should stress that PyBEST uses the Cholesky decomposed integrals and a Tesla V100S, while TeraChem was tested against a Tesla V100. Despite that, we can still make a fair comparison. PyBEST’s CPU–GPU implementation is 3.7 and 2.9 times faster than its CPU counterparts, for the (H_2_O)_10_ and (mU)_2_H_2_O molecule, respectively. Based on the bare GPU times, our CuPy implementation is comparable to the CUDA variant. For the water cluster (H_2_O)_10_ (with fewer basis functions), all operations in PyBEST fit into the GPU VRAM. Thus, the batched variant does not feature any Python-based for loops to distribute the numpy.ndarray to the VRAM. Assuming that a GPU-only CuPy version features similar bottleneck operations like the CPU counterpart, the averaged CuPy-based GPU time would be comparable to the CUDA implementation (assuming that the bottleneck operations take 50% of the total computing time to evaluate the vector function). For the larger system (mU)_2_H_2_O, a batching algorithm must be applied. The batching process utilizes Python-based loops, which are generally slow. Thus, PyBEST’s CPU–GPU drop in performance for the larger system and the increased average GPU time can be attributed to batching. Based on the water cluster calculation, we can anticipate that the batched CuPy implementation, and hence the for loops, introduce a cost factor of approximately 2–4 compared to the CUDA variant.

**Table 3:** Average Timings [s] of a CCSD Iteration Step Compared to Computations with a CUDA Implementation^77^ and Average GPU Time Used in One Iteration^a^

software	AOs	CPU cores	GPU	CCSD time	average GPU time
(H_2_O)_10_, cc-pVDZ, 30 atoms					
Psi4/DF	240	16		16 s	
PyBEST/CD	240	36		337 s	
PyBEST/CD	240	36	Tesla V100S	92 s	4.4 s
TeraChem/CD	240	1	Tesla V100	10 s	10 s
(mU)_2_H_2_O, 6-31+G**, 39 atoms					
PyBEST/CD	468	36		96.5 m	
PyBEST/CD	468	36	Tesla V100S	33.2 m	4.3 m
TeraChem/CD	489	1	Tesla V100	2.5 m	2.5 m

a PyBEST results are shown for CPU and CPU–GPU hybrid variants. CD denotes the Cholesky Decomposition and DF density fitting. The Psi4 data is given as a comparison.

## 7 Conclusions and Outlook

Current trends in scientific programming heavily rely on Python-based implementations,^78^ which are easy to code but need to be more easily scalable to high-performance computing architectures. At the same time, the potential end-users of these codes would like to work on supercomputers to solve problems as large as possible without profound knowledge of high-performance optimizations. To meet those needs for quantum chemistry problems, we analyzed the limitations of quantum chemistry methods written in Python, where some trade-off between the memory and CPU time has to be made. We showed how to use the existing Python libraries to speed up quantum chemical calculations and provided numerical evidence, including comparisons between CPU and GPU.

A common and reasonable practice to speed up an implementation is to identify the so-called bottleneck operations and focus on optimizing these routines. In this work, we focused on the bottlenecks of CCSD-type methods, which are a given set of tensor contractions and their translation into Python code by using various third-party libraries. Specifically, we scrutinized computing timings and memory consumption, comparing numpy.tensordot and opt_einsum calculations. We found that opt_einsum computes about a factor 2.5 faster than a modified version of numpy.tensordot on a CPU but is limited due to tremendous memory peaks and therefore is not a universal candidate when large system sizes are considered. Furthermore, we have rewritten selected routines based on numpy.tensordot, imposing one for loop to prevent the construction of large intermediate arrays. In general, this for-loop implementation is responsible for the drop in efficiency (speed) of numpy.tensordot compared to opt_einsum by approximately a factor of 2. Nonetheless, the gain in memory reduction outweighs the decrease in speed. Although third-party libraries are convenient to interface and easy to use, special attention has to be paid to possible intermediates created during the evaluation of, for instance, tensor contractions. Both opt_einsum and various NumPy linear algebra routines may create several intermediate arrays to increase performance. These memory outbursts may impede a black-box implementation that is computationally feasible for large-scale problems.

A promising alternative for Pythonic large-scale computing is GPUs. We implemented this set of bottleneck tensor contractions to be calculated on the GPU using CuPy, an application programming interface (API) to Nvidia CUDA for Python. Due to the size of the arrays, it is necessary to perform the computations on the GPU batch-wise by splitting the overall tensor contractions into smaller-sized problems that fit into the VRAM. Utilizing this implementation and performing benchmark calculations of the bottleneck contractions showed that a single GPU already leads to a factor of about 10 speed-up compared to our NumPy-based methods using 36 CPU cores. This factor 10 (or 15 in actual production runs) speed-up of the contraction routines only translates to an overall speed-up of factor 3 as the bottleneck of the CC vector function evaluation has shifted to a collection of terms of similar scaling, which are still evaluated on the CPU. Most importantly, our timing benchmark results are encouraging and again prove the potential of GPU utilization in Python-based computational chemistry. The assessment of our Python-based GPU acceleration against a CUDA implementation indicates a drop in performance because of the need for batching due to insufficient GPU VRAM. The anticipated cost of a batched algorithm (or the introduction of for loops in Python) is a factor of 4.

Subsequently, we will identify the “new” bottleneck operations in tensor contractions and export them to the GPU for additional potential performance or a generic GPU implementation using CuPy. Better utilization of the tensor cores could also lead to improvement, as good speed-ups are reported in developer forums, where FP32 input/output data in deep learning frameworks and HPC can be accelerated, running ten times faster than V100 FP32 FMA operations.^79^ Yet, additional speed-up for quantum chemistry problems is expected on the NVIDIA A100 hardware,^80^ which has more GPU cores and up to 80 GB of memory. Generally, direct back-end CUDA implementation in C++ also offers plenty of room for improvements, such as optimization of the array structure or concurrent computing. Alternative routes to export Python code to the GPU are, for instance, offered by the Intel oneAPI toolkits.^81^ Finally, multi-GPU utilization^82^ (for instance, with the NVIDIA NVLink, allowing up to 640 GB of NVRAM) could improve the already tremendous speed-up of factor 10 for tensor contractions.

## Data Availability

The data underlying this study are available in the published article and its Supporting Information and are openly available on Zenodo at https://zenodo.org/records/10069179 and on PyPI at https://pypi.org/project/pybest/.

## Supporting Information Available

Hardware specifications and *xyz* coordinates of the L0 dye relaxed at the B3LYP/cc-pVTZ level of theory.PyBEST inputs/outputs (PDF)
(ZIP)
