# Supplementary material for: Accelerating Pythonic Coupled-Cluster Implementations: A Comparison Between CPUs and GPUs
Source: J Chem Theory Comput. 2024 Feb 2;20(3):1130–42. doi: 10.1021/acs.jctc.3c01110 (PMC10867805; doi:10.1021/acs.jctc.3c01110)
Supplement: Supplementary file 1 — ct3c01110_si_001.pdf [file ct3c01110_si_001.pdf]

Accelerating Pythonic coupled cluster implementations: a comparison between CPUs and GPUs

Maximilian H. Kriebel,* Paweł Tecmer, Marta Gałyńska, Aleksandra Leszczyk, and Katharina Boguslawski*

Institute of Physics, Faculty of Physics, Astronomy and Informatics, Nicolaus Copernicus University in Torun, Grudziadzka 5, 87-100 Torun, Poland

Email: maximilian.kriebel@web.de, k.boguslawski@fizyka.umk.pl

Supplementary Information

S1 Hardware specifications

Node	HPE ProLiant XL190r Gen 10 Server
CPU	Xeon Gold 6240
	36 cores @ 2.6 GHz
	250 GB DDR4-2933 RAM (PC4-23400) @ 23.4 GB/s
Bus	PCIe 3.0 x16
GPU	Nvidia Tesla V100S (rev 1a)
	5120 CUDA cores
	32 GB VRAM

S2 XYZ coordinates of the L0 dye relaxed at the B3LYP/cc-pVTZ level of theory.

C	-0.16014308310542	-0.31199539588878	-2.34547516087214
C	-1.29367892103435	-0.09288979579578	-1.57424913897759
C	0.93809188910984	-0.97698710573475	-1.81020200410545
H	-2.14012582919841	0.43790490632405	-1.98771983276305
H	1.82237386144937	-1.13990028946755	-2.41115941638898
C	-1.34658745592159	-0.55394486292130	-0.25703576187869
C	0.89146139376474	-1.42621187764840	-0.49509337423692
H	1.73911292180745	-1.94632698660134	-0.06896069059724
C	-0.24398995940884	-1.22202545069245	0.27800012927274
H	-0.27966791394245	-1.57601079958710	1.29895671046718
H	-0.13094353646630	0.05205863273511	-3.36395000660610
N	-2.50383774270544	-0.32329941805251	0.54594781401478
C	-3.77958285205046	-0.63221785470791	0.07549561737203
C	-4.91273158259482	0.05914996513168	0.54690651549141
C	-3.97753693874721	-1.64114966125679	-0.88444640438460
H	-4.78920346692929	0.84915309998147	1.27256364054652
H	-3.13251265785560	-2.19850098059872	-1.25990717932935
C	-6.17634739335850	-0.24033049611233	0.08854208715465
C	-5.24489809268507	-1.93254476709909	-1.33872822964226
H	-7.00857883553842	0.32829537530305	0.47225864464343
H	-5.36890543908478	-2.72261714217675	-2.06897278033191
C	-6.38667303716436	-1.25056338436367	-0.87255978098155
C	-2.31532739140353	0.22383217266228	1.85176322083385
C	-2.90392947690499	-0.37945593839548	2.96489536518757
C	-1.51928832059489	1.35663648652690	2.02757824236842
H	-3.51281881810187	-1.26326576728124	2.83347684013940
H	-1.06132715050269	1.82479420079216	1.16727900606923
C	-2.70795173470294	0.15355110879046	4.23192555462058
C	-1.31680780439002	1.87653450328163	3.29926141233491
H	-3.16962811948252	-0.32216462368438	5.08690643512745
H	-0.69955442520667	2.75648576002612	3.42274035862985
C	-1.91272611760180	1.28135078850205	4.40574741602033
H	-1.75796276024297	1.69139042686019	5.39443298578539
C	-7.66035818297096	-1.64260434784333	-1.41749201491158
H	-7.59811537669347	-2.42933622871328	-2.15844944528874
C	-8.93009253110180	-1.21113620168609	-1.17538313654691
C	-10.09427651351423	-1.79072142348142	-1.88570677911231
O	-11.23939014024422	-1.45710178576744	-1.70750977438483
O	-9.75314248521854	-2.75570479974950	-2.78025932826691
H	-10.58430543038918	-3.05592424353792	-3.17382886527890
C	-9.26310405811662	-0.19036704169711	-0.23928916177648
N	-9.51233572895504	0.63782582862546	0.52392463858267
